# Preterm birth impairs postnatal lung development in the neonatal rabbit model

**DOI:** 10.1186/s12931-020-1321-6

**Published:** 2020-02-21

**Authors:** Thomas Salaets, Margo Aertgeerts, André Gie, Janne Vignero, Derek de Winter, Yannick Regin, Julio Jimenez, Greetje Vande Velde, Karel Allegaert, Jan Deprest, Jaan Toelen

**Affiliations:** 1 Department of Development and Regeneration, KULeuven, Herestraat 49, 3000 Leuven, Belgium; 2 Department of Imaging and Pathology, KU Leuven, Leuven, Belgium; 3 Facultad de Medicina, Universidad del Desarollo, Clínica Alemana, Santiago de Chile, Chile; 4 Department of Clinical Pharmacy, Erasmus MC Sophia Children’s Hospital, Rotterdam, The Netherlands; 5 Institute for Women’s Health, University College London Hospital, London, UK

**Keywords:** Bronchopulmonary dysplasia, Prematurity, Preterm birth, Lung development

## Abstract

**Background:**

Bronchopulmonary dysplasia continues to cause important respiratory morbidity throughout life, and new therapies are needed. The common denominator of all BPD cases is preterm birth, however most preclinical research in this area focusses on the effect of hyperoxia or mechanical ventilation. In this study we investigated if and how prematurity affects lung structure and function in neonatal rabbits.

**Methods:**

Pups were delivered on either day 28 or day 31. For each gestational age a group of pups was harvested immediately after birth for lung morphometry and surfactant protein B and C quantification. All other pups were hand raised and harvested on day 4 for the term pups and day 7 for the preterm pups (same corrected age) for lung morphometry, lung function testing and qPCR. A subset of pups underwent microCT and dark field imaging on day 0, 2 and 4 for terms and on day 0, 3, 5 and 7 for preterms.

**Results:**

Preterm pups assessed at birth depicted a more rudimentary lung structure (larger alveoli and thicker septations) and a lower expression of surfactant proteins in comparison to term pups. MicroCT and dark field imaging revealed delayed lung aeration in preterm pups, in comparison to term pups. Preterm birth led to smaller pups, with smaller lungs with a lower alveolar surface area on day 7/day 4. Furthermore, preterm birth affected lung function with increased tissue damping, tissue elastance and resistance and decreased dynamic compliance. Expression of vascular endothelial growth factor (VEGFA) was significantly decreased in preterm pups, however in the absence of structural vascular differences.

**Conclusions:**

Preterm birth affects lung structure and function at birth, but also has persistent effects on the developing lung. This supports the use of a preterm animal model, such as the preterm rabbit, for preclinical research on BPD. Future research that focuses on the identification of pathways that are involved in in-utero lung development and disrupted by pre-term birth, could lead to novel therapeutic strategies for BPD.

## Background

Changing practices in neonatology have altered the phenotype of bronchopulmonary dysplasia (BPD). Antenatal steroids and surfactant supplementation have improved survival of more preterm infants with more immature lungs. This is associated with an increasing incidence of BPD, as defined by oxygen dependency at 36 weeks of life [1]. The adoption of non-invasive ventilation and lower oxygen saturation targets on the other hand have blunted postnatal lung injury. As a result BPD shifted from a fibrotic disease [2] to a disease characterized by alveolar developmental arrest [3].

Despite this evolution to a “new” type of BPD, respiratory disease remains an important long term consequence of preterm birth. For instance, increased rates of respiratory symptoms (e.g. coughing, wheezing, respiratory infections, respiratory drug use) have been reported in survivors of preterm birth [4, 5]. Lung function in survivors of extreme preterm birth is significantly different from term controls at long term, even in the absence of BPD or oxygen dependency [6, 7]**.** Most importantly, a model based on perinatal characteristics such as gestational age, birth weight, sex, race, familial history and intubation at birth is a better predictor for respiratory outcomes than the presence of BPD (as defined by the need for supplementary oxygen) [4]. Further efforts to improve the respiratory outcomes of former preterm neonates thus remain necessary, regardless of oxygen dependency.

Postnatal care (e.g. hyperoxia or mechanical ventilation) can influence respiratory outcome, but the common factor contributing to all BPD cases is prematurity [8, 9]. Nevertheless, most preclinical research on BPD is performed in term neonatal rodents exposed to excessive hyperoxia or aggressive mechanical ventilation [10, 11]. The advantage of rodent models is that terms pups are born in the saccular stage of lung development, morphologically comparable to moderate to very preterm neonates. Functionally however these pups are ready to breathe room air, and they do not exhibit signs of respiratory distress as babies prone to develop BPD do. Overall rodent models lack a premature transition to postnatal life. The translatability of these findings to human “new” BPD can therefore be questioned. Large animal models like preterm baboons and lambs on the other hand can be used to mimic intensive neonatal care with invasive ventilation or even non-invasive pressure support, but impose significant costs [12, 13]. The preterm rabbit model could be an intermediate that combines prematurity and the possibility of perinatal manipulations with relative low cost [8, 14]. Surprisingly, little animal experimental data is available on the persistent effects of a preterm transition to postnatal life, in the absence of any other noxiae, on lung structure and function. Investigating the effect of preterm birth on lung development in animal models however could lead to novel therapeutic strategies for “new” BPD.

In this study we are the first to investigate the effect of prematurity on lung structure and function in neonatal rabbits.

## Methods

### Animal protocol and care

The animalium of KU Leuven provided pregnant rabbits (New Zealand White and Dendermonde hybrid). All experiments were approved by the Ethics Committee for Animal Experimentation (P060/2016 and P080/2017) and were performed according to local guidelines on animal welfare. Does were housed in individual cages with free access to food and water in a controlled 12 h/12 h light-dark cycle. For Cesarean section does were sedated with intramuscular ketamin 35 mg/kg (Nimatek®; Eurovet Animal Health BV, Bladel, The Netherlands) and xylazin 6 mg/kg (XYL-M®; VMD, Arendonk, Belgium). Subsequently they were euthanized with a mixture of 200 mg embutramide, 50 mg mebezonium, and 5 mg tetracain hydrochloride (IV bolus of 1 ml T61®; Intervet Belgium, Mechelen, Belgium). Immediately after death, the abdomen was opened and the uterus exposed to extract all pups through hysterotomy. After birth, pups were dried, stimulated and placed in an incubator (Dräger Incubator 7310; Dräger, Lübeck, Germany) at 32 °C, 50% relative humidity and 21% of oxygen. Pups were fed twice daily via an orogastric tube with a milk replacer (Day One®, Protein 30%, Fat 50%; FoxValley, Illinois, US) with additional probiotics, electrolytes and vitamins (Bio-Lapis®; Probiotics International Ltd., Somerset, UK) and immunoglobulins during the first 2 days (Col-o-Cat®, SanoBest, Hertogenbosch, Netherlands). The volume of feeds is increased from a total of 80 ml/kg body weight/day on day 0 of life to 200 ml/kg/day on days 3–7. On day 2, vitamin K1 is administered intramuscularly (0.25 mg/kg, Konakion pediatrique®; Roche, Basel, Switzerland), and from day 2 onwards, pups are given a daily intramuscular injection of benzylpenicillin (20,000 IU/kg Penicilline®; Kela, Sint-Niklaas, Belgium) and amikacin (20 mg/kg day, Amukin®; Bristol-Myers-Squibb). MicroCT and dark field imaging was performed under isoflurane anesthesia (2% isoflurane in 2 l/min of pure oxygen). Lung functions were terminal experiments performed in deeply anesthetized postnatal rabbit pups (ketamine 35 mg/kg and xylazin 6 mg/kg IM). Anesthetized animals were euthanized by exsanguination for dissection and lung harvest.

### Study groups

**Fig. 1:**
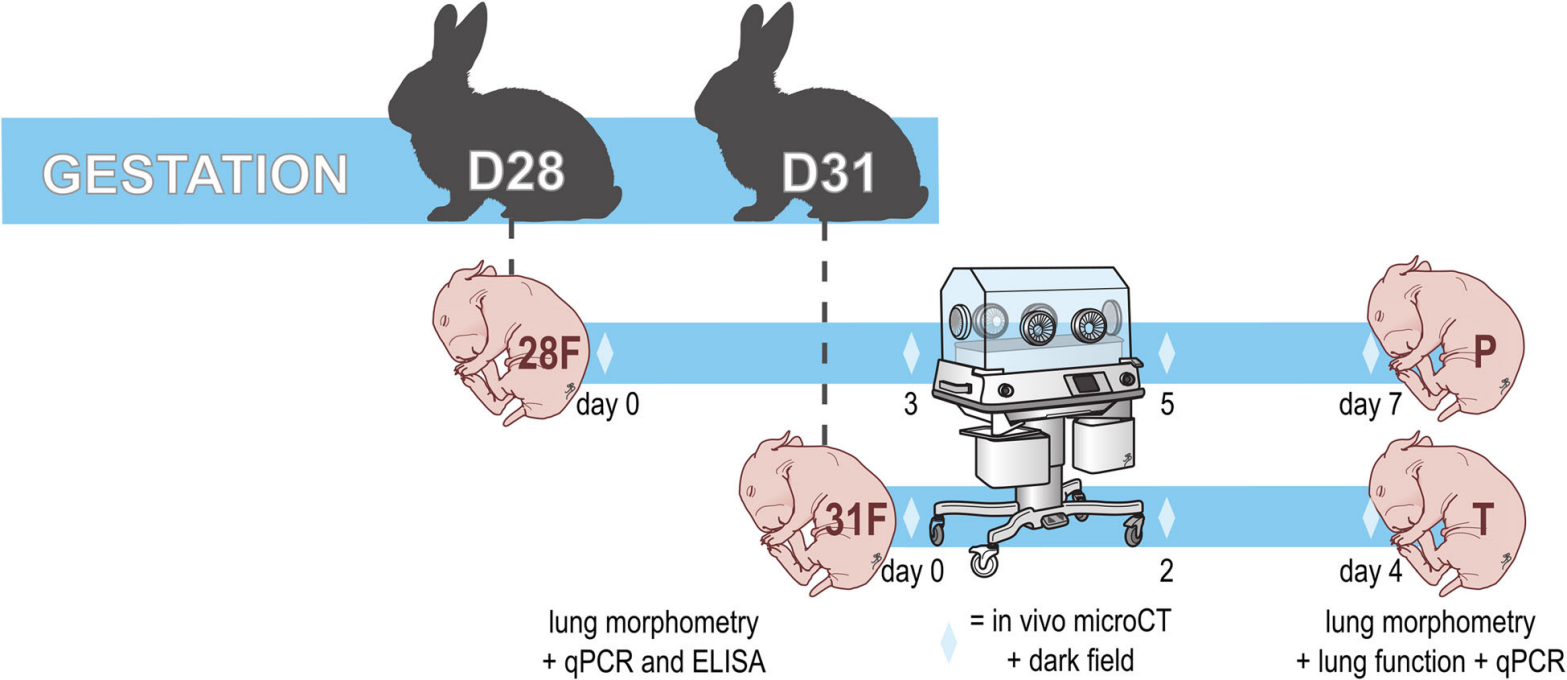
Timeline of the rabbit experiments. *Pups were delivered on either day 28 or day 31. For each gestational age pups were harvested (n = 10–15) immediately after birth, before the first breath: 28F and 31F. All other pups (n = 15–25) were hand reared and harvested on day 4 for the term pups (T) and day 7 for the preterm pups (P; same corrected age). A subset of pups underwent microCT and dark field imaging on day 0, 2 and 4 for terms and on day 0, 3, 5 and 7 for preterms*

The study groups and timeline are illustrated in Fig. 1. Pregnant dams were randomized to either a Cesarean section on day 31 (term, alveolar stage of lung development) or day 28 (preterm, saccular stage). Of each group, randomly selected pups were harvested before their first breath: they constitute the fetal groups 28F (preterm, day 28) and 31F (term, day 31). In fetal pups left lungs were harvested for histology, and right lungs snap frozen for molecular analysis. The other pups were stimulated to breath by tactile stimulation. Pups surviving the first adaptation hour after birth were hand reared until day 7 (preterm pups) and day 4 (term pups; same corrected age): they constitute the postnatal groups P (preterm) and T (term). A subset of the postnatal pups underwent in vivo microCT and dark field imaging on day 0, day 3, day 5 and day 7 in P and on day 0, day 2 and day 4 in T (same corrected ages). On day 7/day 4, all postnatal pups underwent lung function testing before snap freezing of the right lung, and left lung harvest for histology.

### Histological read-outs

Left lungs were harvested for histology, and therefore pressure fixed on 25cmH_2_O of paraformaldehyde for 24 h. Volume of fixed left lungs was measured by water immersion (Scherle’s principle). Lungs were embedded in paraffine, and for every lung a central sagittal 5 μm slide was made for hematoxylin-eosin staining. Stained sections were scanned with a slide scanner (Axio Scan® Slide Scanner, Zen Zeiss, Oberkochen, Germany). Alveolar morphometry measurements are semi-automatically performed with a self-designed Fiji-plugin on 20 randomly selected fields per lung. Based on the counts mean linear intercept (Lm, estimating alveolar size) and mean transsectional wall length (Lmw, estimating septal wall thickness), and total alveolar surface (S, by taking the reference left lung volume in to account) were calculated [15, 16].

A second central 5 μm slide was stained with Miller’s elastic staining. For at least 15 arteries of 30-100 μm in diameter, the internal and external diameter of the medial-layer was determined on the shortest axis. Average relative thickness of the medial layer (MT%) was calculated per animal as described before [16]. On a third slide an immunohistochemical staining of CD31 was performed in order to assess vascular density. Tissue preparation for immunohistochemistry included deparaffinization, heat retrieval, endogenous blockage of peroxidase (0.5% H_2_O_2_ in methanol) and protein blockage. Endothelial cells were stained with a mouse anti-human CD31 antibody at 10.25 μg/ml (M0823, DakoCytomation). A secondary goat anti-mouse antibody (Z0420, DakoCytomation) was used in combination with an alkaline phosphatase anti-alkaline phosphatase antibody (STAR67, Bio-Rad, Hercules, CA, USA) and nitroblue tetrazolium as a chromogen. We manually counted vascular structures of 20-50 μm on 5 randomly selected high power fields (hpf) of 500x500μm.

Additionally, one slide per lung was stained with a Sirius Red collagen staining. The amount of collagen was estimated by automatic measurement of the stained area on 20 randomly selected lung fields (500μmx500μm) relative to the whole field.

### mRNA expression of surfactant proteins

mRNA was extracted from snap frozen right lung tissue using the Tri-Pure® Isolation reagent (Roche Diagnostics, Germany). RNA concentration was measured using the Nanodrop 1000® spectrophotometer (Thermo Scientific) and RNA integrity was assessed by checking the integrity of the 18S and 28S band by agarose gel electrophoresis. cDNA was synthesized with Taq Man® Reverse Transcription Reagents (ThermoFisher Scientific). The Platinum SYBR® Green qPCR Supermix-UDG with ROX (ThermoFisher Scientific) was used to detect expression of surfactant protein B and C, elastin, collagen 1A2 (COL1A2) and vascular endothelial growth factor (VEGFA). YWHAZ, HPRT and actin B were used as stable housekeeping genes to normalize mRNA levels. An overview of used primers, obtained from Integrated DNA Technologies (Haasrode, Belgium), can be found in Additional file 1: Table S1. Samples were run in triplicate on a StepOne Plus® instrument (ThermoFisher Scientific). Relative quantitation was determined using the comparative Ct method. Statistics are calculated in based on ΔΔCt-values, while fold change (FC) is used for visualization.

### Surfactant protein B ELISA

Snap frozen right lung tissue was homogenized according to the manufacturer’s instructions, in a protein lysis buffer containing Tris-HCL and EDTA. Surfactant protein B was quantified using a commercial ELISA kit (MBS2601137, MyBioSource, San Diego, California, USA).

### Sex determination

For a subset of animals, a piece of skin was snap frozen for sex determination through a PCR of the specific region of Y-chromosome (SRY). DNA was isolated using the GenUP gDNA Kit (Biotechrabbit, Henningsdorf, Germany). The SRY fragment was amplified using PCR, and the obtained PCR products were analyzed by gel electrophoresis. In all samples rabbit GAPDH was used as an internal amplification control. Primers are listed in additional file 1: Table S1.

### Lung function testing

FlexiVent 5.2 (SCIREQ, Montreal, Canada) is used to perform pulmonary function tests on deeply anesthetized postnatal pups. The trachea of the pup is surgically exposed and an 18G metal cannula is inserted in the trachea and connected to the FlexiVent ventilator (120 breaths/min ventilation; tidal volume of 8 ml/kg). A pressure volume perturbation is performed to measure inspiratory capacity and static compliance. Snapshot is a single-wave forced oscillation perturbation used to assess dynamic compliance, elastance and resistance from a single compartment model. Finally, Prime-8 is a broadband forced oscillation technique and is used to assess tissue damping (frequency independent parenchymal energy dissipation) and tissue elastance (energy conservation of lung tissue). Three measures for each perturbation are obtained per pup and the mean value is used in the analysis. Measures for compliance, elastance and inspiratory capacity were corrected for weight. After lung function, deeply anesthetized animals are euthanized by exsanguination.

### Micro computed tomography (microCT)

Rabbit pups were fixated on a Styrofoam bed in the supine position in a low-dose small animal microCT scanner (SkyScan 1278, Bruker micro-CT, Kontich, Belgium). The following acquisition parameters were used: 1 mm Al filter, 50 kVp X-ray source voltage, 918 μA source current, 55 ms exposure time, 9 projection images per 0.9° rotation step over a total angle of 180°. Respiratory gating was performed retrospectively by matching projection images to the signal of a camera registering breathing movements. A 3D datasets with a 50 μm isotropic reconstructed voxel size was created. We used software provided by the manufacturer (TSort, NRecon, DataViewer and CTan) to gate, reconstruct, visualize and analyze microCT data [17]. End-expiratory data is used for analysis.

Lung volume was manually delineated on all slices of the reconstructed microCT dataset, avoiding the inclusion of the heart and mediastinal structures, by a researcher (MA) blinded for group allocation. Total (end-expiratory) lung volume and mean lung density were calculated. Additionally, by applying a fixed density threshold (85 on a grayscale from 0 to 255), aerated lung tissue was segmented from the non-aerated lung tissue. Aerated and non-aerated lung volume was calculated as a percentage of the total lung volume. To calibrate mean lung density for Hounsfield units (HU), a phantom (air-filled tube of 1.5 mL inside a water-filled tube of 50 mL) was scanned [18]. Only the outcomes of the pups that survived until day 7 were used for the analysis (*n* = 7–9).

### Dark field imaging

Dark field imaging is a form of phase-contrast x-ray imaging, and was performed on a Talbot-Lau interferometer with the pup held in a straight position on a Plexiglas mold. The interferometer operated at 40 kVp with a 2 μm G2 grating period and a system visibility of 22%. The radiation dose per round was 0.51 mGy [19, 20]. From the acquisitions, dark field images were reconstructed through a MATLAB-algorithm. The intensity of the dark field signal in 4 quadrants of every lung image was scored on a scale from 0 (no signal indicating little aeration) to 8 (strongest signal indicating complete aeration) by 3 independent observers who were blinded for the group allocation. The average score of the 4 quadrants was considered representative for the animal, and the average of the 3 observers is reported.

### Statistical analysis

GraphPad Prism® 7.0 software (GraphPad, La Jolla, California, USA) was used for statistical analysis. Normality was assumed after visual inspection of the data. Statistical outliers were removed from the analysis if a Grubbs test was positive. All values are expressed as mean ± standard deviation. Unpaired t-testing with Welch correction for unequal variance was used to compare outcome measures in 28F to 31F and P to T. A mixed-effects model was used to compare the longitudinal outcomes between the different time points. Bonferroni-Sidak correction for multiple comparisons was used to compare groups on individual time points. Kaplan–Meier curves with post hoc log-rank testing were used to quantify survival of rabbit pups from fetal day 28 until postnatal day 7 (preterm) or 4 (term), assuming that intrauterine deaths in the term mothers occurred before day 28 (*n* = 3). A *p*-value of < 0.05 was considered statistically significant.

## Results

### Fetal rabbits on day 28 exhibit functional and structural immaturity

**Fig. 2:**
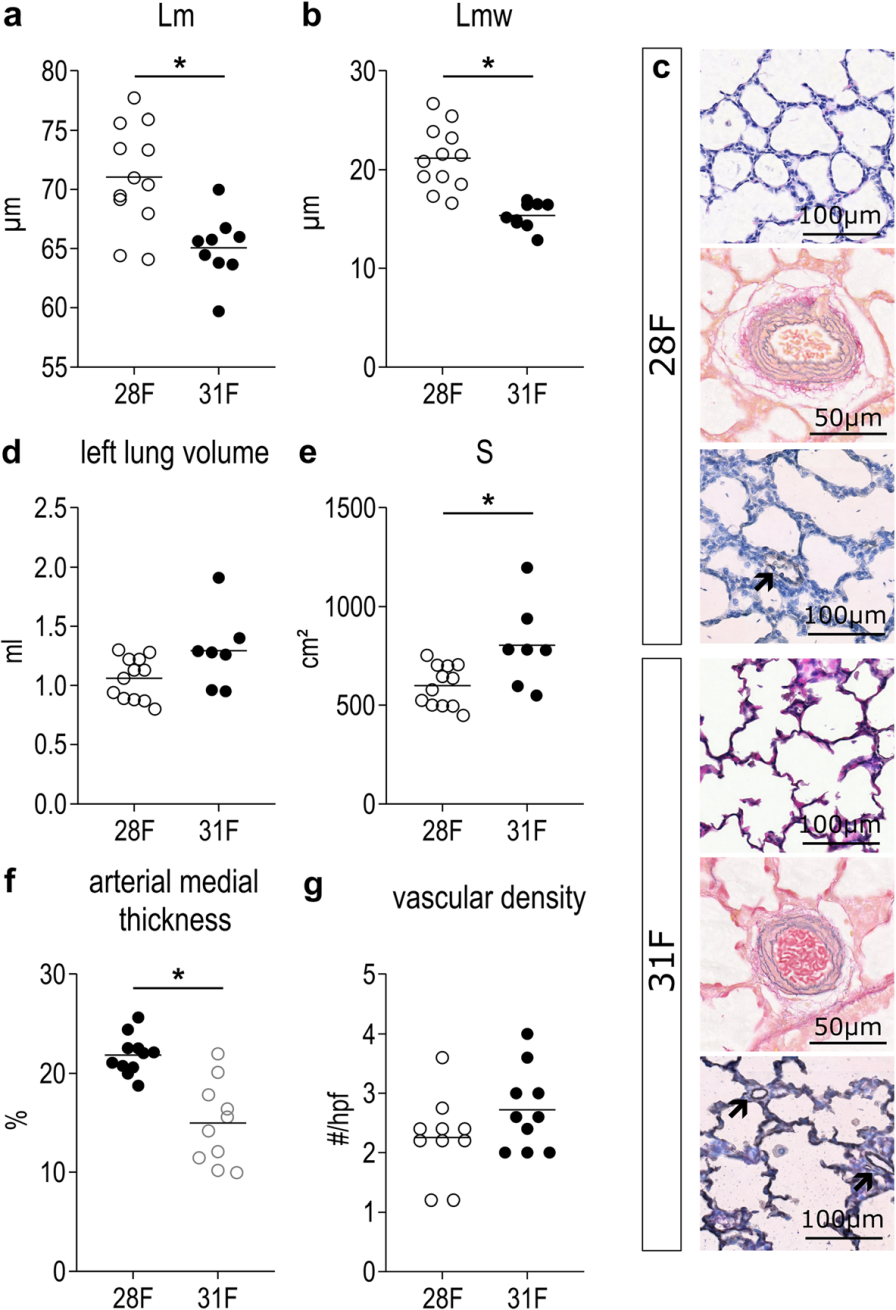
Structural and functional immaturity of rabbit pups born on day 28 of gestation. ***a*** *Lm and (****b****) Lmw are measurements of alveolar size and septal thickness respectively and are both increased in preterm pups at birth.* ***c*** *Representative pictures of lung slides stained with HE,Miller and CD31 indicate more rounded and less complex airspaces, more muscularized arteries and a comparable density of small to medium size vessels in preterm pups at birth. Arrows indicate 20-50* μm *vessels with CD31-positive lining.* ***d*** *Left lung volume, as measured by water displacement, is not significantly different, however* ***e*** *total alveolar surface is significantly decreased in preterm pups, however proportionate to the decreased body size.* ***f*** *Arterial medial thickness is higher in preterm pups at birth.* ***g*** The number of small to medium size vessels per high power field is comparable in term and preterm pups at birth. *p < 0.05

**Fig. 3:**
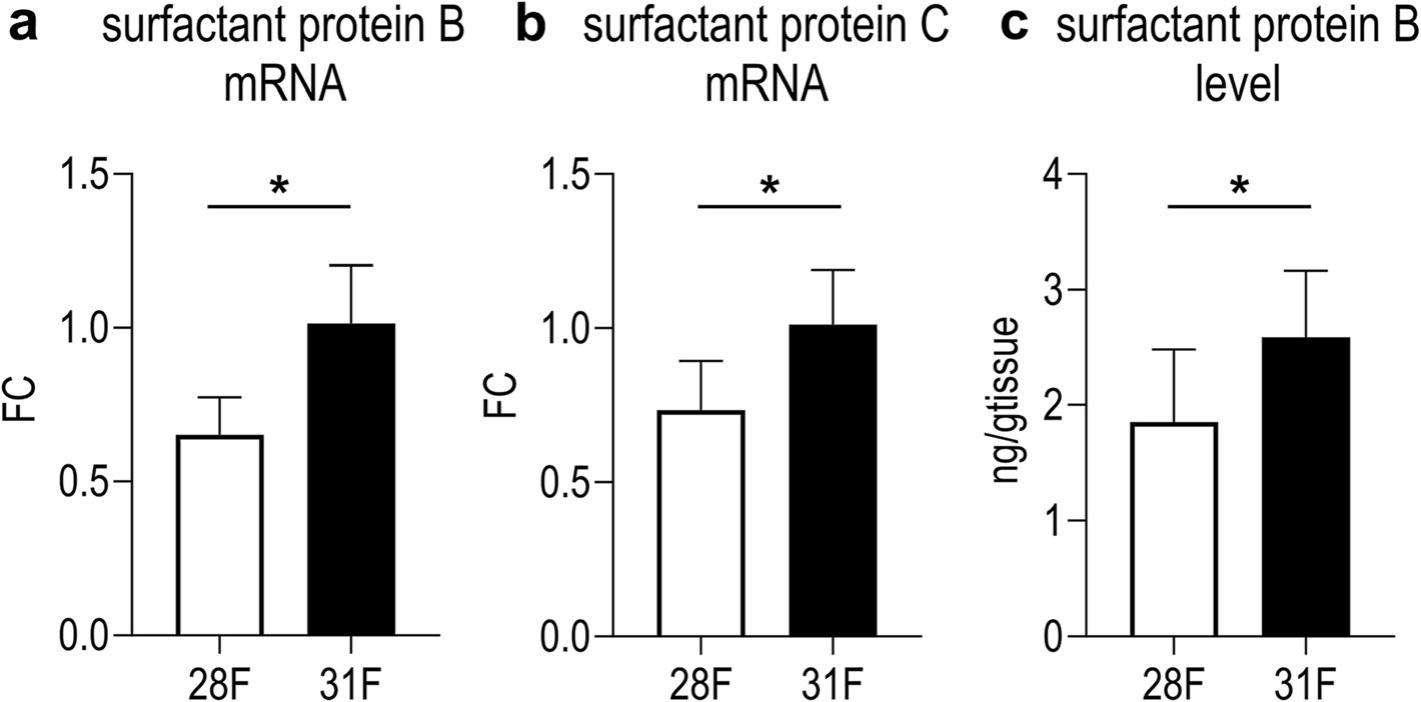
Surfactant expression in term and preterm pups at birth. ***a****,* ***b*** *Surfactant protein B and C mRNA expression is significantly lower in preterm pups before the first breath (n = 5).* ***c*** The level of surfactant protein B in lung homogenate is lower in preterm pups, compared to term pups (n = 10–15). *p < 0.05

Fifteen preterm and 10 term fetal animals were harvested before the first breath, from respectively 5 and 3 mothers. Birth weight was significantly lower in 28F than in 31F (*p* = 0.0109; Additional file 2: Table S2). Lungs of preterm rabbits had a significantly higher Lm (*p* = 0.0012) and an increased Lmw (*p* < 0.0001) compared with term fetuses (Fig. 2a, b). The representative figures depict the more rounded and less complex airspaces, with a higher cellularity in 28F (Fig. 2c). Left lung volume was not significantly lower in 28F (*p* = 0.2927), but due to the simplified lung structure total alveolar surface (S) is significantly decreased (*p* = 0.0484; Fig. 2d, e). However, the difference in alveolar surface is proportional to the difference in birth weight (*p* = 0.5162 if alveolar surface is normalized to weight). Additionally, arterial medial thickness was higher in 28F compared to 31F (*p* = 0.0004; Fig. 2f); representative pictures in Fig. 2c. Vascular density however was not significantly different between 28F and 31F (*p* = 0.1500; Fig. 2g); respresentative pictures in Fig. 2c. Finally, preterm fetuses also demonstrate lower mRNA-expression of surfactant protein B and C than term fetuses (*p* = 0.0055 and *p* = 0.0282 respectively; Fig. 3a, b). Also at a protein level, there was a decreased amount of surfactant protein B present in the fetal lung tissue of 28F-pups, in comparison to 31F-pups (*p* = 0.0065; Fig. 3c).

### Preterm rabbit pups have higher mortality and lower weight than term controls

**Fig. 4:**
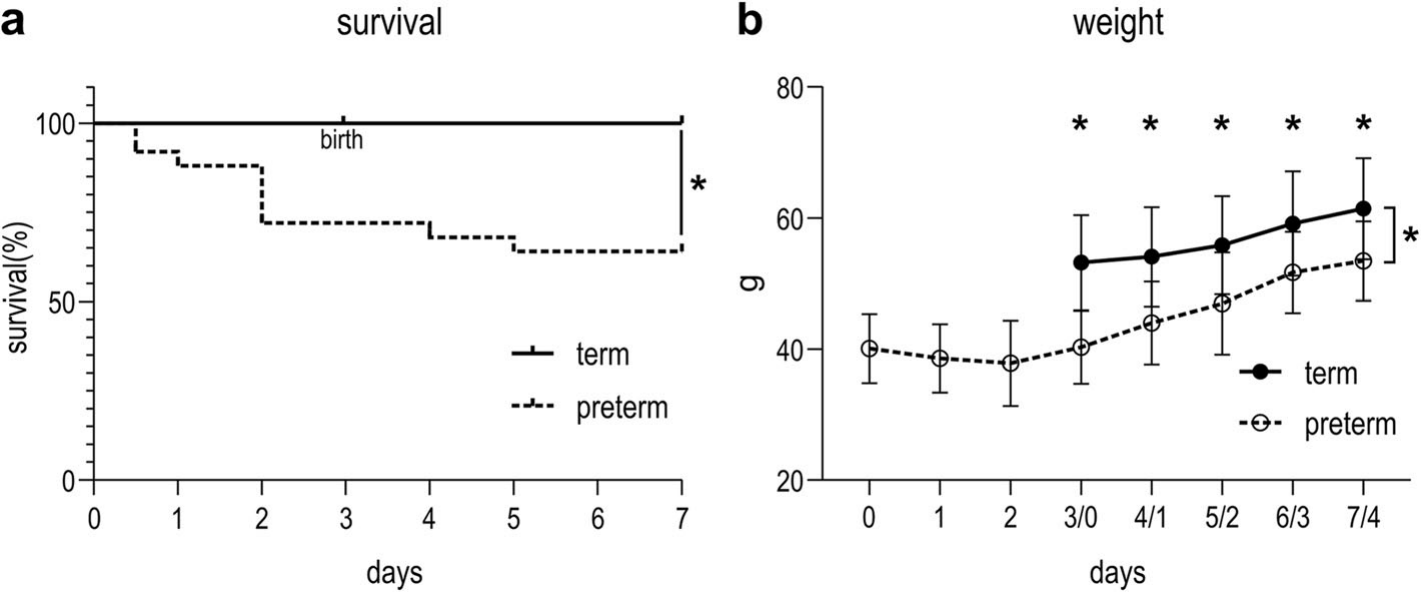
Survival and weight of term and preterm rabbit pups. ***a*** *Survival was significantly lower in pups born preterm (60%; 15/25) then in animals born at term (100%; 15/15).* ***b*** At every time point, preterm pups were significantly smaller than term controls. *p < 0.05

Four mothers were randomized to term and 8 to preterm delivery. In the first hour after birth, 100% (47/47) of term pups and 55.8% (43/77) of preterm pups survived the adaptation to postnatal life. Fifteen term and 25 preterm randomly selected pups were included in the experiment. Survival was significantly lower in pups born preterm (60%; 15/25) then in animals born at term (100%; 15/15; *p* = 0.0105; Fig. 4a). Over time, weight evolved significantly different between preterm and term animals (*p* = 0.0005). On day 7/day 4 (day of harvest), preterm pups were significantly smaller than term counterparts (*p* = 0.0190; Fig. 4b; Additional file 2: Table S2).

### Preterm rabbit pups demonstrate delayed lung aeration

**Fig. 5:**
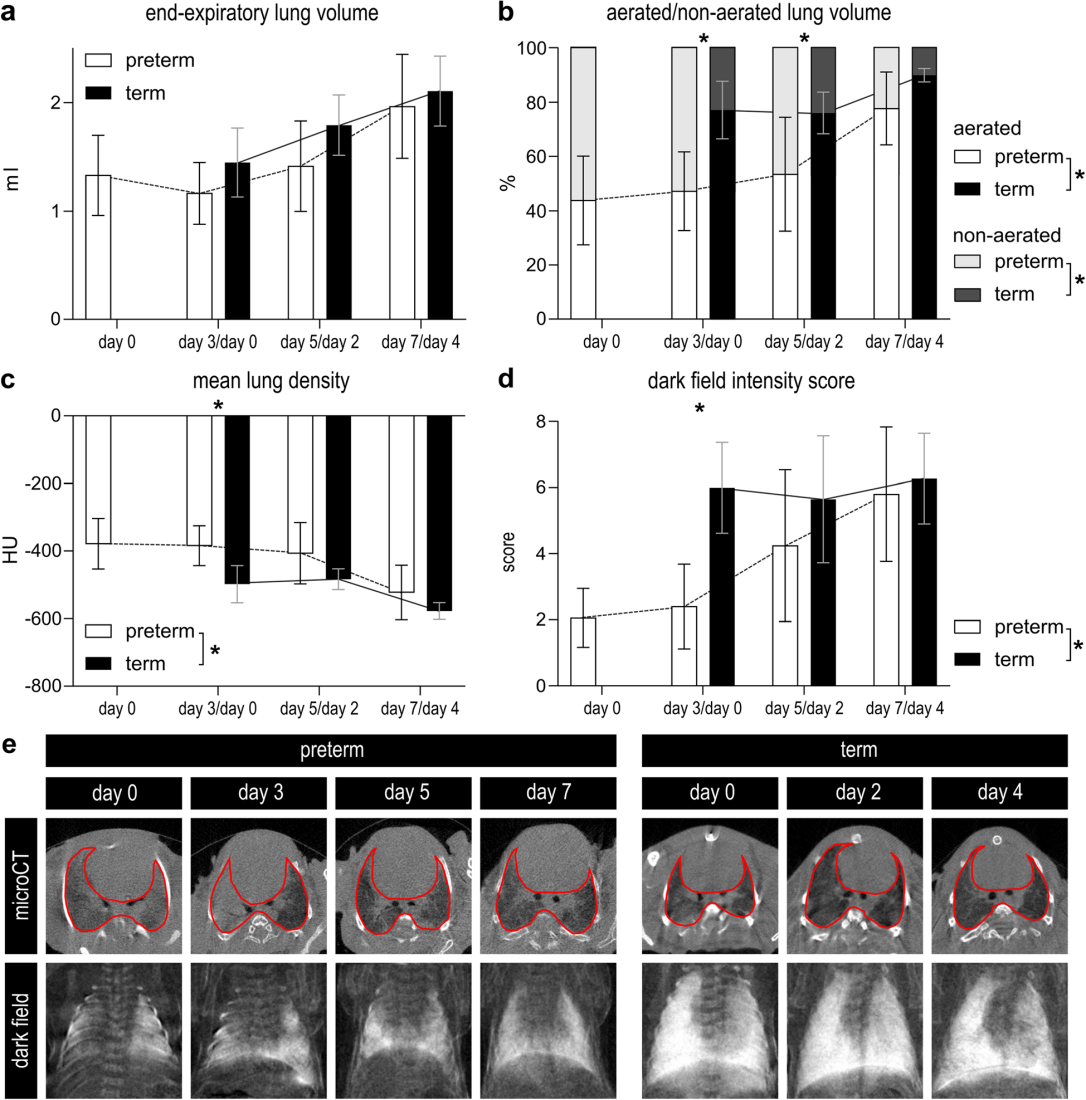
In vivo imaging of lungs of term and preterm animals. ***a*** *Total lung volume assessed by microCT increases over time in both term and preterm pups (n = 7–9).* ***b*** *Preterm pups have a lower proportion of aerated lung volume then term pups on day 3/day 0 and on day 5/day 2 (n = 7–9; assessed by microCT).* ***c*** *Preterm pups have lower mean lung density then term pups on day 3/day 0 (n = 7–9; assessed by microCT; expressed in Hounsfield units (HU)).* ***d*** *Dark field score is significantly lower in term animals* versus *preterm animals on day 3/day 0.* ***e*** Representative pictures of transversal sections of microCT volumes and dark field images in preterm and term animals on all time points. *p < 0.05

In vivo microCT-derived end-expiratory lung volume grew significantly over time in both the preterm and term animals (*p* < 0.0001). Lung volume was not significantly different in both groups (*p* = 0.1297; Fig. 5a). Additionally, the proportion of aerated lung volume changed over time in both P and T (p < 0.0001). Lungs of pups in P had a significantly lower proportion of aerated lung volume (*p* = 0.0001), with significant posthoc differences on day 3/day 0 and day 5/day 2, and a strong trend towards less aerated volume on 7/day 4 (*p* = 0.0022, *p* = 0.0282 and *p* = 0.0504 respectively). Conversely, non-aerated lung volume decreased over time and was significantly larger in P (Fig. 5b). Consequently, also the mean lung voxel density decreased with time (p < 0.0001), with a generally significantly higher density in P compared to T (*p* = 0.0008). Posthoc testing only revealed a significant difference on day 3/day 0 (*p* = 0.0085; Fig. 5c). At visual analysis, lungs of preterm pups, especially in the early time points but also later, were characterized by atelectatic lung regions, diffuse ground glass areas and sometimes air bronchograms. Representative images of the transversal microCT-sections can be found in Fig. 5e.

In line with the microCT-results also the dark field intensity scores, reflecting lung aeration, increased over time in the preterm group (*p* = 0.0016), but not in the term animals (*p* = 0.6723) that obtained high scores already immediately after birth. Dark field intensity was overall higher in T in comparison to P (*p* = 0.013), however at posthoc comparisons the difference was only significant on day 3/day 0 (*p* < 0.0001; Fig. 5d). Representative dark field images can be found in Fig. 5e.

### Preterm birth has persistent effects on lung volume and function

**Fig. 6:**
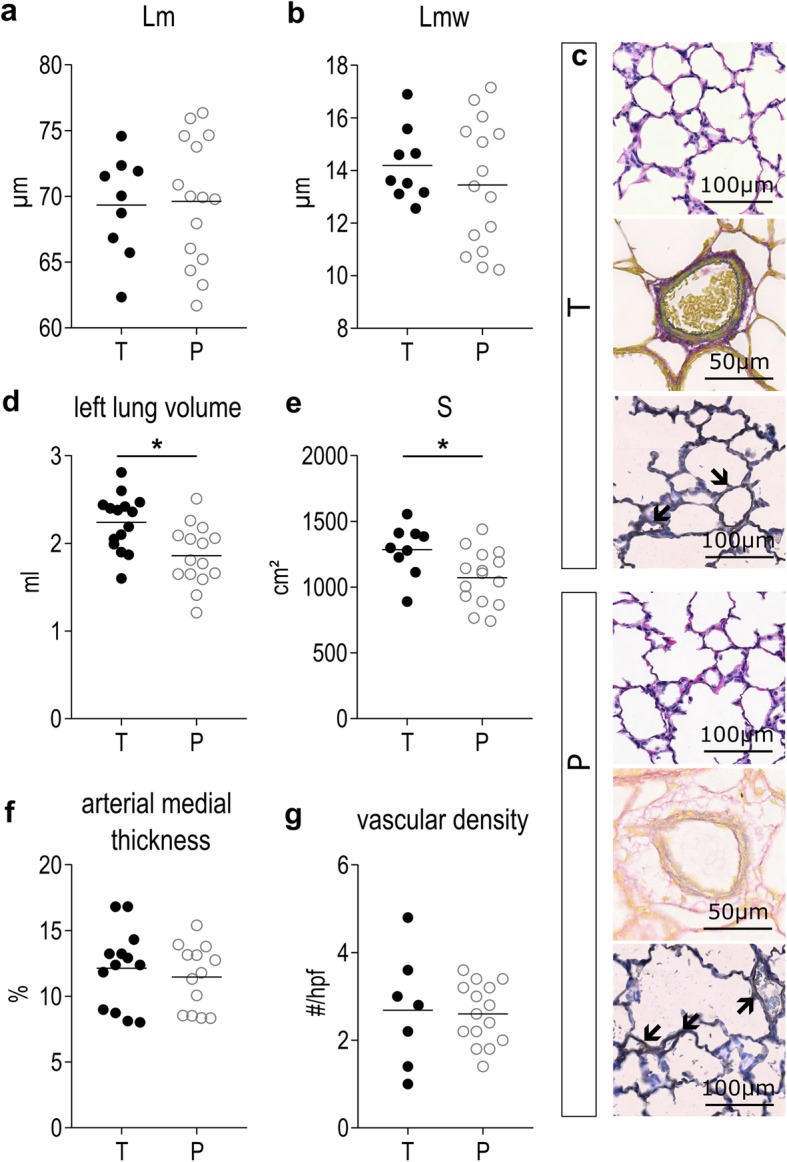
Effects of prematurity on alveolar and vascular structure. ***a*** *Lm and (****b****) Lmw are not affected by preterm birth.* ***c*** *Representative pictures of lung slides stained with HE, Miller and CD31 indicate comparable alveolar structure, comparable arterial wall thickness and comparable vascular density in both groups. Arrows indicate 20-50 μm vessels with CD31-positive lining.* ***d*** *Left lung volume is smaller in preterm animals.* ***e*** *Total alveolar surface is smaller in preterm animals compared to term controls, however proportionate to the smaller body weight.* ***f*** *Arterial medial thickness is not affected by preterm birth.* ***g*** The number of small to medium size vessels per high power field is not affected by preterm birth. *p < 0.05

**Fig. 7:**
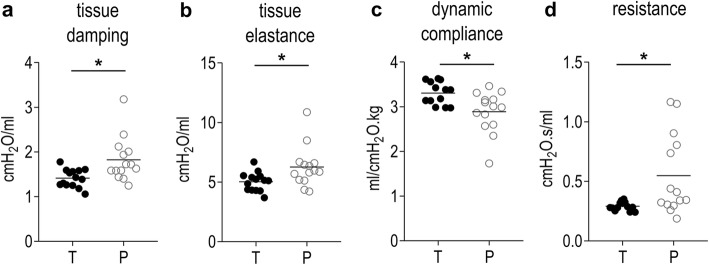
Effects of prematurity on lung function. ***a****-****d*** *Lung function read-outs tissue damping, tissue elastance, dynamic compliance and resistance are significantly affected by preterm birth. Other lung function read-outs can be found in* Additional file 3: Table S3. *p < 0.05

Seven days after birth, Lm and Lmw were not significantly different in preterm pups (P), versus term pups at the same corrected age (T; Fig. 6a-c). Because ex vivo left lung volumes were significantly smaller in P than T (*p* = 0.0042; Fig. 6d), total alveolar surface area (S) was decreased in P versus T (*p* = 0.0210; Fig. 6e). The difference in alveolar surface area was proportional to the difference in body weights of the groups (*p* = 0.3231 if alveolar surface is normalized to weight). Arterial medial wall thickness was not significantly different between P and T (*p* = 0.5386; Fig. 6c and f). Additionally; vascular density was comparable in P and T (*p* = 0.8740; Fig. 6c and g). Tissue mechanics were altered in preterm animals, with increased tissue damping and tissue elastance in P (*p* = 0.0104 and *p* = 0.0240 respectively; Fig. 7a, b). Airway resistance tended to be higher in P (*p* = 0.1008; Additional file 3: Table S3). At single wave oscillation, dynamic compliance corrected for weight was increased in P (*p* = 0.0079; Fig. 7c). Consequently, dynamic elastance corrected for weight was decreased in P (*p* = 0.0159; Additional file 3: Table S3). Resistance was also significantly higher in P (*p* = 0.0140; Fig. 7d). In pressure-volume perturbations, static compliance and elastance, and inspiratory capacity (all corrected for weight), were not significantly different (Additional file 3: Table S3).

**Fig. 8:**
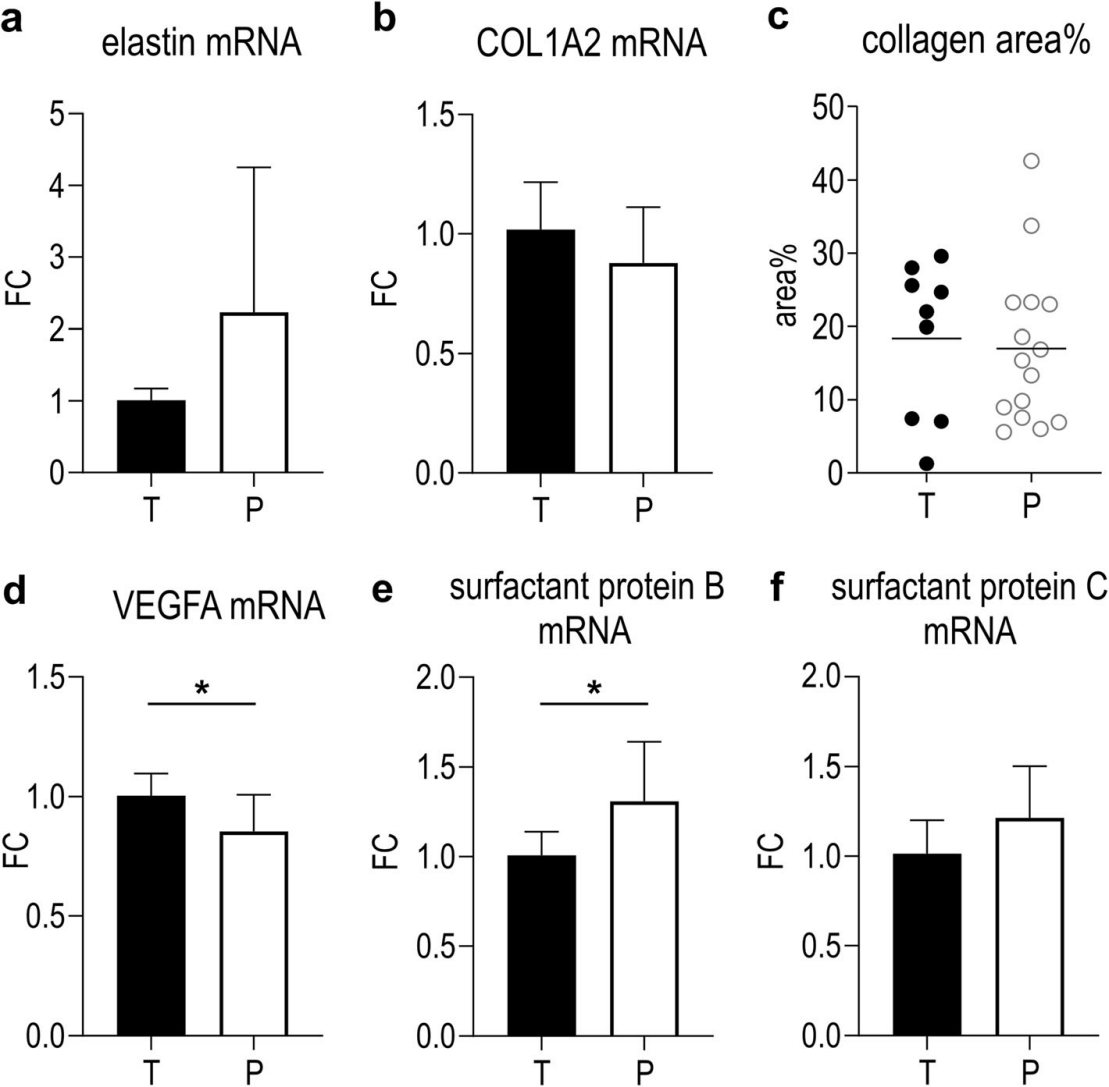
Effects of prematurity on extracellular matrix components, VEGFA and surfactant proteins. **a** Elastin mRNA-expression is ambiguously dysregulated by preterm birth (n = 10). **b**, **c** Nor COL1A2 mRNA-expression (n = 10) nor the collagen positive area on lung tissue slides is significantly dysregulated by preterm birth. **d** VEGFA mRNA-expression is significantly decreased after preterm birth (n = 10). **e**, **f** Surfactant protein B and C mRNA-expression is upregulated by preterm birth (n = 10). *p < 0.05

We observed no significant changes in mRNA-expression of extracellular matrix components elastin and collagen 1A2 in P versus T (*p* = 0.1382 and *p* = 0.1593; Fig. 8a, b). However there was a marked variation in elastin mRNA expression in P. We also did not observe any significant change in collagen positive area on lung tissue slides (*p* = 0.7574; Fig. 8c). VEGFA mRNA-expression was significantly decreased in P in comparison to T (*p* = 0.0173; Fig. 8d). Finally, surfactant protein mRNA-expression was also increased in P versus T: significantly for surfactant protein B (*p* = 0.0089; Fig. 8e), but only a trend for surfactant protein C (*p* = 0.0733; Fig. 8f).

There were no significant differences between male and female preterm pups for the main outcomes of this experiment, however it was not powered for this comparison (Additional file 3: Figure S1).

## Discussion

In this study we investigated the effect of preterm birth on lung development in preterm rabbits. In preterm fetal rabbits, harvested at birth, we demonstrated structural immaturity. Thicker septa with a higher cellularity and less complex airspaces indicate that preterm pups at day 28 of gestation are in the saccular stage of lung development. This observation is in line with early descriptive work on lung development in rabbits [21]. Furthermore preterm fetuses exhibited thicker arterial walls, indicating higher pulmonary vascular resistance. We also observed lower surfactant protein B and C mRNA expression and surfactant protein B levels compared with term animals, indicating functional prematurity. Additionally we noted significantly decreased lung aeration on microCT and dark field imaging, in comparison to term pups. Together with earlier described maturational lung function changes in preterm rabbits at birth [22], this suggests preterm rabbits exhibit a phenotype comparable to respiratory distress syndrome (RDS). In the absence of respiratory support this resulted in increased work of breathing and apnea, with a mortality of 44,2% in the first hour of life. This proves the structural and functional immaturity of preterm rabbits born on day 28.

The primary objective of this study was to assess whether birth with immature lungs affects further lung development. By performing in vivo microCT and dark field imaging we established that preterm pups demonstrate delayed lung aeration, requiring at least 7 days before reaching recruitment comparable to term pups. Despite birth in a saccular stage of lung development, after 7 days the alveolar structure of pups born preterm was comparable to that of term pups on day 4, indicating continued postnatal lung development. However, because lungs of (smaller) preterm pups were smaller in size, they had lower total alveolar surface area. It is relevant to note here that preterm pups did not catch-up with the weight of the term pups, within the time course of the experiments, and that the difference in alveolar surface area was proportionate to the difference in weight. Despite the catch-up of structural lung development (relative to body weight), lung function on corrected day 4 was significantly affected with altered tissue mechanics, dynamic compliance and elastance It is unclear whether this relates to the marked but ambiguous dysregulation of elastin mRNA-expression (which is reminiscent of changes in elastic tissue in tissue from infants with BPD [23]) or to the (possibly compensatory) upregulation of surfactant protein mRNA-expression. Finally, there was a decreased expression of VEGFA-mRNA in preterm pups, in absence of any structural vascular differences. A decreased expression of VEGFA is an important finding, given the crucial role of this molecule in lung development [24, 25]. We hypothesize that, in this mildly sick model, altering mainly the alveolar stage of lung development, structural vascular changes might be limited to the capillary bed in the alveolar walls, which is hard to quantify (and often understudied).

Only a few research groups so far have investigated the effect of a precocious adaptation of immature lungs to postnatal life on further lung development. Experiments in preterm sheep and baboons indicate that preterm birth together with mechanical ventilation results in an alveolar developmental arrest [12, 13]. However, when ventilator induced lung injury was reduced by the use of non-invasive respiratory support, the difference between preterm and term lungs disappeared [26, 27]. In contrast, our data suggests that even in the absence of respiratory support and prenatal injury (e.g. chorio-amnionitis), preterm birth affects lung development in rabbits and results in a mild lung disease phenotype. Many studies have looked at different etiological factors involved in the pathogenesis of BPD, such as hyperoxia of ventilator induced lung injury, but to our knowledge, this is the first study that focuses on the effect of preterm birth alone on lung development.

We postulate three possible explanations for the effects of preterm birth on the developing lung. Even in the absence of hyperoxia, oxidative stress might play an important role in arresting lung development after preterm birth. Room air contains a supraphysiological oxygen pressure in comparison to the uterine environment, and preterm lungs have weaker anti-oxidant defense mechanisms [28, 29]. Supraphysiological oxygen concentrations might disrupt normal expression of oxygen-sensitive molecules such as VEGFA [24, 30]. Recently it has also been shown that even room air affects the behavior of fetal lung mesenchymal stem cells [31]. Another possible explanation is related to nurture and growth. With twice daily gavage feedings using a specific rabbit formula, we were not able to match in utero growth. This is however comparable to the clinical setting, where neonatologists are also not able to match the nutritional requirements of the fetus as closely as the placenta can in utero. Digestive immaturity, a deficit in specific nutritional components and the absence of placental or maternal growth factors might be involved in the altered body and lung development of the preterm pups [32]. The latter hypothesis backs the experimental therapy with recombinant insulin-like growth factor 1 (IGF1) [33], and could also explain the observed decrease in VEGFA mRNA-expression. Finally, altered lung function might also be explained by atelectasis or delayed lung fluid clearance in preterm lungs, as suggested by the microCT imaging. Our biochemical data however suggests that surfactant mRNA-expression catches up and is compensatory high in pups born prematurely.

Our study has several limitations. First, the time frame of the study is limited. In the absence of a mother it is challenging to make rabbits pups thrive longer than 1 week. It is possible that lung development of preterm pups would catch-up if studied over a longer period of time. Second, the rabbit pups in this study were, despite proven structural and functional immaturity, born only 3 days (or 10% of gestational age) before term. Decreasing the gestational age of the animals might increase the effect on lung development. The high perinatal mortality at day 28 and our unpublished experiences with pups born at day 27, suggest however that day 28 is the limit of viability, in the absence of respiratory support measures. It should be noted that this mortality in the preterm group induces a selection bias, with only data of the stronger survivors included in the outcome parameters. Any study investigating the effect of preterm birth will have to balance maximal immaturity, with an acceptable survival rate. Adding surfactant administration or respiratory support measures might increase survival and enhance translatability, but can mask the effect of preterm birth alone. Third, the model lacks prenatal insults. On day 28 we deliver healthy preterm rabbits, while in humans prematurity rarely comes alone. Pre-eclampsia or chorio-amnionitis do not only predispose for preterm birth, but also affect lung development in utero as part of a multiple hit concept [34, 35]. The lack of a prenatal insult however could also be a strength, as this allows us to study the individual effect of prematurity.

As stated in the introduction, the phenotype of BPD in clinics has changed significantly. Furthermore, recent data suggest that long term respiratory outcome is affected in survivors of extreme preterm birth, regardless of a formal BPD diagnosis [4–6]. Even late preterm birth has been shown to affect lung function later in life [36, 37]. At a functional level, clinical experiments with forced oscillation techniques comparable to those used in this paper, showed worse results in former preterm infants compared to term controls [38, 39]. In general, pre- and postnatal factors (e.g. infection, oxygen, volutrauma) can contribute to the development of post-prematurity respiratory disease or BPD, however the one common denominator is preterm birth itself [8, 9]. The findings in this study support the idea that preterm birth alone, even in the absence of any other pre- and postnatal injury, affects lung development.

Translation of findings from BPD animal models to new BPD in clinics has been difficult in the past [40]. A possible explanation can be the lack of functional prematurity in commonly used rodent models for BPD [10, 11]. The knowledge from this study asks for a paradigm shift in BPD research, focusing less on hyperoxia and ventilator induced injury, and more on the effect of prematurity. Large animal models such as preterm lambs and baboons mimic the structural and functional immaturity of an extremely preterm neonate, however practical and ethical constraints limit extensive use [12, 13]. The preterm rabbit model could be an elegant compromise between the presence of prematurity and ease of experimentation [8, 14]. Our finding that preterm birth alone affects developing lungs, supports the use of structurally and functionally preterm animal models for the study of BPD, such as rabbits born on day 28 of gestation.

Future research initiatives should focus on disentangling the physiological processes and molecular pathways involved in the effect of preterm birth on lung development (e.g. transcriptome analysis in preterm versus term lungs). Additionally, more research is needed to identify the growth factors and processes that are essential for normal in utero lung development in the second and third trimester. Insight in these mechanisms could lead to novel therapeutic strategies to improve the respiratory outcomes of survivors of preterm birth.

## Conclusions

In this paper we demonstrated that rabbit pups born on day 28 exhibit structural and functional immaturity at birth. Preterm birth impairs normal lung development and results in a functional, and to a lesser extent structural, deficit at 1 week after birth. Our results suggest that prematurity itself causes a lung developmental delay, even in the absence of hyperoxia or mechanical ventilation. Future research that focuses on the identification of pathways that are involved in in-utero lung development and disrupted by pre-term birth, could lead to novel therapeutic strategies for BPD.

## Supplementary information

**Additional file 1: Table S1.** Primer sequences used for qPCR in this study.

**Additional file 2: Table S2.** Weight and survival data of the pups used in this study.

**Additional file 3: Table S3.** Lung function data.

**Additional file 4: Figure S1.** Comparison of female (*n* = 5) and male (*n* = 3) preterm rabbit pups. Included female pups weighed 47.6 ± 4.9 g and included male pups weighed 53.1 ± 3.2 g (mean ± sd) at birth. There was no significant difference in weight gain. (A-H) There were no significant differences in the main study outcomes between male and female preterm pups. *p < 0.05.

## Data Availability

The datasets used and/or analysed during the current study are available from the corresponding author on reasonable request.

## Abbreviations

28F: Fetal preterm group (born on day 28 of gestation)
31F: Fetal term group (born on day 31 of gestation)
BPD: Bronchopulmonary dysplasia
COL1A2: Collagen 1A2
FC: Fold change
hpf: High power field
HU: Hounsfield units
IGF1: Insulin-like growth factor 1
Lm: Mean linear intercept
Lmw: Mean transsectional wall length
microCT: Micro computed tomography
MT%: Relative thickness of the medial layer
P: Postnatal preterm group (born on day 28 of gestation, harvested on day 7)
RDS: Respiratory distress syndrome
S: Total alveolar surface area
T: Postnatal term group (born on day 31 of gestation, harvested on day 4)
VEGFA: Vascular endothelial growth factor A
