# Supplementary material for: Preterm birth impairs postnatal lung development in the neonatal rabbit model
Source: Respir Res. 2020 Feb 21;21:59. doi: 10.1186/s12931-020-1321-6 (PMC7035772; doi:10.1186/s12931-020-1321-6)
Supplement: Supplementary file 1 — Additional file 1: Table S1. Primer sequences used for qPCR in this study. [file 12931_2020_1321_MOESM1_ESM.docx]

Table S1. Primer sequences used for qPCR in this study.

| **Table S1. Primer sequences.** | |
| --- | --- |
| surfactant protein B FW | ACTGTCTACAGGAAGTCTG |
| surfactant protein B RV | GAAGTAGACGTCAAGCACG |
| surfactant protein C FW | CAAAGAGGCCTTGATGGAGA |
| surfactant protein C RV | ATCTCTAGGACCATCTCGGT |
| elastin FW | AGCCAAATACGGTGCTGCT |
| elastin RV | CACCTGGGTAAATGGGAGAC |
| collagen 1A2 FW | TGCAGGGCTCCAATGATGTT |
| collagen 1A2 RV | AGAATTCTTGGTCAGCGCCA |
| vascular endothelial growth factor A FW | CTTGCTGCTCTACCTCCACC |
| vascular endothelial growth factor A RV | CTTTGGTCTGCATTCACATTTG |
| YWHAZ FW | TGATTAGTGATGATGAACCG |
| YWHAZ RV | CACACAGAGGGCTACAATG |
| HPRT FW | GGTCTGGCCCTTAACTTCTCTGTGTTCTA |
| HPRT RV | GCGTGCTGTCTTTGTATGATTCTTCACTT |
| actin B FW | CAAGCGTGGCATCCTGAC |
| actin B RV | CTCGTTGTAGAAGGTGTGGTG |
| specific region of Y chromosome FW | AGCGGCCAGGAACGGGTCAAG |
| specific region of Y chromosome RV | CCTTCCGGCGAGGTCTGTACTTG |
| GAPDH FW | TCACCATCTTCCAGGAGCGA |
| GAPDH RV | CACAATGCCGAAGTGGTCGT |
