# Supplementary material for: Preterm birth impairs postnatal lung development in the neonatal rabbit model
Source: Respir Res. 2020 Feb 21;21:59. doi: 10.1186/s12931-020-1321-6 (PMC7035772; doi:10.1186/s12931-020-1321-6)
Supplement: Supplementary file 2 — Additional file 2: Table S2. Weight and survival data of the pups used in this study. [file 12931_2020_1321_MOESM2_ESM.docx]

Table S2. Weight and survival data of the pups used in this study.

| **Table S2. Baseline characteristics.** | **Preterm fetal**  **28F** | **Term fetal**  **31F** | **Term**  **T** | **Preterm**  **P** |
| --- | --- | --- | --- | --- |
|  | mean ± sd | mean ± sd | mean ± sd | mean ± sd |
| **Day 0** |  |  |  |  |
| Number of pups (n) | 15 | 10 | 15 | 25 |
| Number of mothers (n) | 5 | 3 | 4 | 8 |
| Birth weight (g) | 36.9±4.6 | 47.9±10.7 | 53.2±7.3 | 40.1±5.3 |
| **Day 7** |  |  |  |  |
| Survival (n, %) | - | - | 15 (100%) | 15 (60%) |
| Harvest weight (g) | - | - | 61.5±7.7 | 53.5±6.0 |
