# Supplementary material for: Preterm birth impairs postnatal lung development in the neonatal rabbit model
Source: Respir Res. 2020 Feb 21;21:59. doi: 10.1186/s12931-020-1321-6 (PMC7035772; doi:10.1186/s12931-020-1321-6)
Supplement: Supplementary file 3 — Additional file 3: Table S3. Lung function data. [file 12931_2020_1321_MOESM3_ESM.docx]

Table S3. Lung function data.

| **Supplementary Table 3. Lung function data.** | **Term**  **T** | **Preterm**  **P** | |
| --- | --- | --- | --- |
|  | mean ± sd | mean ± sd | *p-value* |
| **Static measures** |  |  |  |
| Inspiratory capacity (ml/kg) | 62.7±5.2 | 61.2±7.9 | *0.5486* |
| Static compliance (ml/cmH_2_O.kg) | 2.2±0.5 | 2.5±0.7 | *0.2488* |
| Static elastance (cmH_2_O.kg/ml) | 0.48±0.10 | 0.42±0.12 | *0.3236* |
| **Forced oscillation** |  |  |  |
| Tissue damping (cmH_2_O/ml) | 1.4±0.2 | 1.8±0.5 | *0.0104* |
| Tissue elastance (cmH_2_O/ml) | 5.0±0.8 | 6.3±1.7 | *0.0240* |
| Airway resistance (cmH_2_O.s/ml) | 0.13±0.04 | 0.21±0.17 | *0.1008* |
| **Single frequency oscillation** |  |  |  |
| Dynamic compliance (ml/cmH_2_O.kg) | 3.3±0.3 | 2.9±0.5 | *0.0079* |
| Dynamic elastance (cmH_2_O.kg/ml) | 0.30±0.03 | 0.36±0.08 | *0.0159* |
| Resistance (cmH2O.s/mL) | 0.29±0.04 | 0.55±0.34 | *0.0140* |
