# Supplementary material for: Preterm birth impairs postnatal lung development in the neonatal rabbit model
Source: Respir Res. 2020 Feb 21;21:59. doi: 10.1186/s12931-020-1321-6 (PMC7035772; doi:10.1186/s12931-020-1321-6)

**Figure S1.** Comparison of female (n=5) and male (n=3) preterm rabbit pups. Included female pups weighed 47.6±4.9g and included male pups weighed 53.1±3.2g (mean±sd) at birth. There was no significant difference in weight gain. (A-H) There were no significant differences in the main study outcomes between male and female preterm pups, however this explorative analysis is not sufficiently powered. **p<0.05*


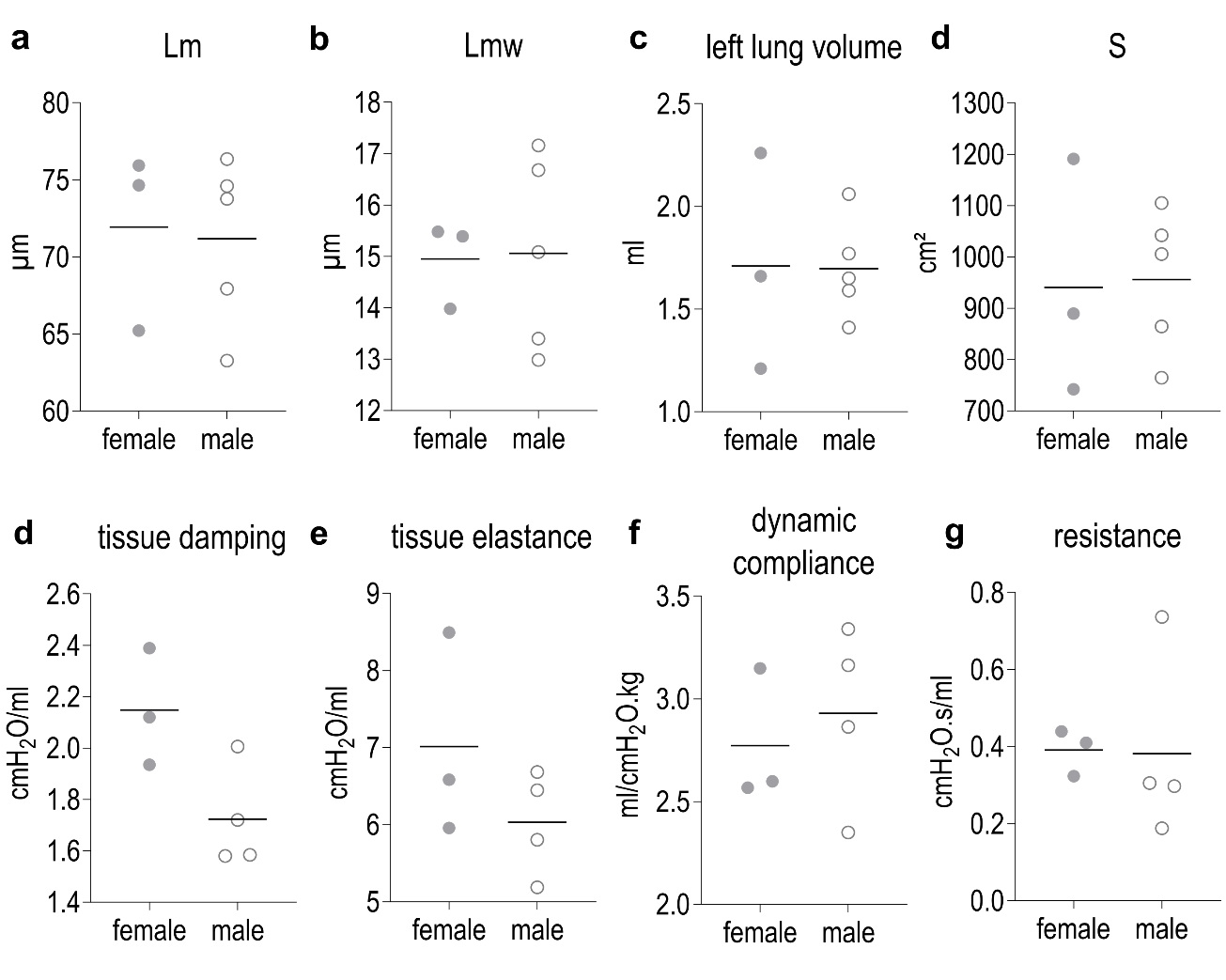

Supplement: Supplementary file 4 — Additional file 4: Figure S1. Comparison of female (n = 5) and male (n = 3) preterm rabbit pups. Included female pups weighed 47.6 ± 4.9 g and included male pups weighed 53.1 ± 3.2 g (mean ± sd) at birth. There was no significant difference in weight gain. (A-H) There were no significant differences in the main study outcomes between male and female preterm pups. *p < 0.05. [file 12931_2020_1321_MOESM4_ESM.docx]
